# Environmental physiology research presented at ICEE2013

**DOI:** 10.1186/2046-7648-2-22

**Published:** 2013-07-01

**Authors:** James David Cotter, Samuel John Edwin Lucas, Toby Mündel

**Affiliations:** 1 School of Physical Education, Sport and Exercise Sciences, University of Otago, P.O. Box, 56, Dunedin 9054, New Zealand; 2 School of Sport and Exercise Sciences, University of Birmingham, Birmingham B15 2TT, UK; 3 School of Sport and Exercise, Massey University, Private Bag 11 222, Palmerston North 4442, New Zealand

**Keywords:** Heat Stress, Cold, Exercise, Hypoxia, Adaptation, Climate Change, Space, Physiology, Comfort

## Abstract

The 15th International Conference on Environmental Ergonomics, Queenstown, New Zealand, February 11 to 15, 2013 (ICEE2013) brought together researchers interested in work and exercise physiology, safety, comfort and performance in various stressful and extreme environments.

## What is ICEE?

Environmental ergonomics focuses on humans in stressful environments (especially heat, cold, altitude, diving and space) often from integrative or applied perspectives and in relation to work or exercise. The International Conference on Environmental Ergonomics (ICEE) meetings also provide a valuable forum for those working in whole-body integrative human science more generally. Important features of ICEE meetings, which run single-stream over 4 to 5 days, are the collegiality, the collective knowledge of many long-term attendees, and the strong ethos on fostering the development of student researchers (with assistance from WL Gore & Associates since 2007). The evolution of the ICEE is described elsewhere (e.g., Mekjavić, Taylor and Di Prampero; Eur J Appl Physiol 104: 127–129, 2008). From the meeting's inception in 1984, a well-represented stress is that of excessive thermal energy, as caused by high metabolic production, oppressive microclimates (clothing or equipment) or wider environments. This year's meeting was no exception, with many heat stress presentations, ranging from acute and adaptive physiology, heat illness, psychophysiology and cognition, ergonomics and comfort, thermal modelling, clothing and climate. The present report highlights some environmental physiology research presented within ICEE2013, for which peer-reviewed abstracts or mini papers are available (http://www.environmental-ergonomics.org).

## ICEE2013

In the opening sessions, *climate change* and *occupational heat stress*, Tord Kjellström (Umea University, Sweden) emphasised that increasing climatic and factory ambient temperatures are disproportionately impacting on the health and productivity of the world's most disadvantaged workers and economies. Kjellström's group also summarised an open, international research programme (High Occupational Temperature: Health and Productivity Suppression) and detailed online, worldwide meteorological data available to researchers. They demonstrated that the highest relative excess mortality in hot periods occurs among outdoor workers (while the elderly continue to have the highest absolute rates of mortality). Whereas, preliminary modelling from The Netherlands (Hein Daanen, TNO, The Netherlands) indicates that global warming will *reduce* that country's mortality and morbidity and associated costs (i.e., less cold-related mortality), but in financial terms, these benefits will be outweighed by lost productivity in midsummer. Excessive heat stress was reported from quantitative and qualitative studies in workplaces ranging from Korean agriculture to Danish kitchens and New Zealand shearing sheds. Similarly, more prevalent extreme high-temperature days were projected from meteorological analyses for Poland and USA, with productivity loss estimated to increase by 1% p.a. in regions of the USA. Problems due to an increased reliance on air conditioning were addressed in several presentations. Nigel Taylor's group (University of Wollongong, Australia) showed that personal bushfire shelters might maintain physiological strain below critical limits for exposures up to 1 h. All highlighted the need for thermal physiologists to engage more actively in finding energy-efficient solutions to the problems of increasing heat in urban and rural workplaces.

New research on *water immersion* centred mostly on thermal stress. Chris Button's group (University of Otago, New Zealand) found faster recovery of respiratory cold-shock responses in experienced swimmers than novices when treading water of 10°C, but comparable cold-induced reductions in swimming capacity and modest cerebrovascular impacts. In a follow-up field study, swimming capacity was more than halved in open water (14°C vs. 27°C pool), but swimmers also estimated their capacity conservatively. Kerri Ann Evely (Memorial University, Canada) showed that wearing a dive mask doubled volunteers' breath-hold capacity with facial-only immersion in 0°C water, due entirely to the lesser cold shock. We reported that competitive-intensity endurance swimming in warm water (30°C to 32°C, i.e. comfortable *T*_sk_) did not cause hyperthermia or a risk of insidious hyperthermia, whereas cold intolerance was prevalent when swimming in cool water (20°C).

Blinded *hypohydration* of 2% and 3% body mass did not impair trained cyclists' time-trial performance in the heat (Paul Laursen, AUT University, New Zealand), in agreement with recent meta-analyses by Goulet (Br J Sports Med 2013;47:679-686). Similarly, cognition was not impaired at 3% and 5% hypohydration, with or without hyperthermia; in fact, perceptual performance was *improved* (Anne van den Heuvel, University of Wollongong, Australia). Nathan Morris (University of Ottawa, Canada) elegantly demonstrated a role of gastric thermoafferent input to sudomotor control, which might also help account for previous studies showing that ingesting crushed ice facilitates higher terminal core temperatures being tolerated in heat-stressful performance tests.

Anthony Bain (UBC Okanagan, Canada) provided a detailed partitioning of cerebrovascular responses to *severe heat strain*; facial cutaneous perfusion did not steal from cerebral perfusion, but rather heat-induced hypocapnia could account for the lower perfusion to the entire brain. Yuta Hoshi (University of Tsukuba, Japan) found that people can voluntarily suppress this hyperventilation and thereby preserve eucapnia and cerebral perfusion. Bain and Hoshi won first prize for best student oral and poster presentations, respectively. Julien Périard (Aspetar, Qatar, and University of Sydney, Australia) and Sebastian Racinais (Aspetar, Qatar) confirmed that heat strain *per se* causes central fatigue, but exercise *per se* tended to superimpose a peripheral fatigue, which is partly offset by heat-induced improvement in neuromuscular function.

Other highlights of original research on *acute and chronic heat stress* included the following findings: Mechanoreflex input to cardiovascular and sudomotor control is independent of the level of metaboreflex input (Tatsuro Amano, Kobe University, Japan); downward modulation of exercise intensity due to high skin temperatures seems to occur for prolonged, but not short endurance exercise (Koen Levels, TNO, The Netherlands); exercise-related improvement in expiratory function is at least partly due to elevated lung temperature (Pippa Band, University of Portsmouth, UK); the rise in core (rectal) temperature was again found to be related more closely to heat production per unit mass than to %$\dot{V}O_{2}\text{max}$ (Matthew Cramer and Ollie Jay, University of Ottawa, Canada); neither L-menthol nor compression tights had clear effects on exercising thermoregulation or performance (Martin Barwood, University of Portsmouth, UK); sweat efficiency is higher in females than in males due to minimal sweat drippage (Yoshimitsu Inoue, Osaka International University, Japan); and tropical indigenes retain thermal perceptive, but not thermoafferent autonomic heat adaptations during prolonged (>2 years) residence in a temperate climate (Joo-Young Lee, Seoul National University, Korea) and reacclimatise during summertime, further indicating that their thermoregulatory benefits are adaptive more than genetic (Titis Wijayanto, Kyushu University, Japan).

More generally, Taylor's group presented a very extensive analysis of the interactions of core, skin and local skin temperatures in the *control of skin blood flow* in different acral and non-acral skin regions during hypothermia, normothermia and hyperthermia. Hanns-Christian Gunga (Charité Medical University, Germany) showed promising validation data for measuring core temperature using a non-invasive ‘double sensor’, in extremes ranging from bed rest to space travel to exercise. Shawnda Morrison and Igor Mekjavić (Jozef Stefan Institute, Slovenia) have undertaken *combined-stressor studies* of interactions between chronic hypoxia and chronic activity level (bed rest, sedentary and daily exercise) on sleep, cardiovascular, thermoregulatory, musculoskeletal and functional outcomes. Short-term (10 days) hypoxia generally did not exacerbate effects of unloading nor did exercise alter those of hypoxia. Similarly, cold and hypoxia were shown to additively mediate fatigue of muscular endurance (Alex Lloyd, Loughborough University, UK). A session on *novel stress conditioning* was premised on the fact that discrete stressors (all of which are contained within exercise) can drive adaptations independently and interdependently. For example, mechanical tension, oxidative stress and heat can stimulate adaptations for both health and functional capabilities; these can be imposed selectively using different forms of exercise or environmental (including nutritional) exposure.

Other papers of potential interest to this readership are on ultra-endurance exercise, thermoregulation and aging, cold stress, and predictive accuracy of thermal manikin studies and modelling. The next ICEE meeting will be closer to home for many: Portsmouth, UK, provisionally June 28 to July 3, 2015, organised by Mike Tipton and Jim House.

## Competing interests

The authors declare that they have no competing interests.

## Authors' contributions

JC drafted this report, but SL and TM provided critical feedback. All three authors actively comprised the local scientific committee of ICEE2013 and provided critical review feedback to authors on their abstracts and papers, most of which were subsequently accepted for inclusion in the programme and conference proceedings. All authors read and approved the final manuscript.

